# Bench study of automated tube compensation versus pressure support after extubation in icu patients

**DOI:** 10.1186/2197-425X-3-S1-A278

**Published:** 2015-10-01

**Authors:** L Baboi, H Penet, A Stoian, H Yonis, F Gobert, R Tapponnier, F Bayle, V Leray, J-C Richard, C Guérin

**Affiliations:** Hopital de la Croix Rousse, Lyon, France; INSERM UMR 955, Créteil, France

## Introduction

Automated tube compensation (ATC) is a new mode of ventilatory assistance that aims to compensate for the resistance of endotracheal tube and, hence to reduce the resistive work of breathing.

## Objectives

The aim of this study was to measure triggering system and pressurization in pressure support ventilation with and without ATC.

## Methods

Once removed the endotracheal tube from patients, who were extubated in our ICU after successful weaning trial, was connected to a filter (Hygrobac), and both were attached to ASL 5000 active servo lung (IngMar medical). The lung model was set with a compliance of 50 ml/cmH2O and resistance of 10 cm H2O/L/s. The inspiratory effort included sinusoidal half-wave shape, muscular pressure -10 cmH2O, respiratory rate 27 breaths/min. The ventilator used was Evita XL (Dräger) set as used in patients during the weaning trial in our ICU, ie pressure support 7 cm H2O above PEEP of 4 cm H2O. ATC was set to 80% compensation and the internal diameter of the endotracheal tube for each patient was entered into the ventilator dialog box. Pressure support was run for 2 minutes without then with ATC. Pressure time product (PTP) trigger was defined as the product of airway pressure to time spent between onset of inspiratory effort and pressurization. The quality of airway pressurization was assessed as the area of airway pressure over time from inspiratory effort to 0.5 s later (PTP500). PTP500 was further expressed as percent of ideal PTP500, which was airway pressure recorded at the end of inspiratory time multiplied by 0.5 s. The values are expressed as median [first-third quartiles]. The comparisons were made by Wilcoxon signed rank test with continuity correction.

## Results

We included 40 patients (29 men), aged 71 [62-80] years, SAPS2 on ICU admission 50 [43-62]. They have been intubated for 5 [2-8] days before extubation. The internal diameter of the endotracheal tube was 7.0 mm in 4 patients, 7.5 in 33 and 8.0 in 3. The results pertaining to PTP are displayed in the table. As shown, PTP trigger was significantly lower and PTP500 significantly higher with than without ATC for similar pressure support level.

**Figure 1:**
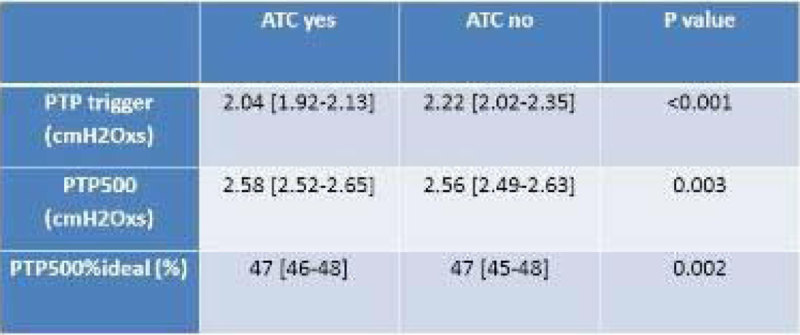
Figure 1

## Conclusions

ATC provided with efficient compensation for used endotracheal tube. The clinical significance of these results remains to be done.

